# How does the spin-state of Co ions affect the insulator-metal transition in Bi_2_A_2_Co_2_O_8_ (A = Ca, Sr, Ba)?

**DOI:** 10.1038/srep38212

**Published:** 2016-11-30

**Authors:** Xiaokun Huang, Weiyi Zhang

**Affiliations:** 1 National Laboratory of Solid State Microstructures and Department of Physics, Nanjing University, Nanjing, 210093, China; 2 Collaborative Innovation Center of Advanced Microstructures, Nanjing University, Nanjing, 210093, China

## Abstract

The misfit layered Bi_2_A_2_Co_2_O_8_ (A = Ca, Sr, Ba) compounds experience an insulator to metal transition as A’s ionic radius increases. This feature is contradictory to the conventional wisdom that larger lattice constant favors insulating rather than metallic state, and is also difficult to be reconciled using the Anderson weak localization theory. In this paper, we show from the first-principles calculation that an insulator-metal transition takes place from a nonmagnetic low-spin state of Co^3+^ ions to a hexagonally arranged intermediate-spin low-spin mixed-state in CoO_2_ plane when ionic radius increases from Ca to Ba. The predicted low-spin state of Bi_2_Ca_2_Co_2_O_8_ and Bi_2_Sr_2_Co_2_O_8_ and intermediate-spin low-spin mixed-state of Bi_2_Ba_2_Co_2_O_8_ are consistent not only with their measured transport properties, but also with the magnetic-field suppressed specific-heat peak observed at the transition temperature. In agreement with experiments, strong electronic correlation is required to stabilize the low-spin insulator and intermediate-spin low-spin metal.

Describing the microscopic behavior of strongly correlated electronic systems in transition-metal oxides with geometrically frustrated lattice structures is a fundamental challenge^1^,^2^. Strong correlations coexist with magnetic frustration, orbital degeneracy, and charge-ordering effects often lead to novel physical phenomena^3^,^4^. For examples, unconventional superconductivity was discovered in water-intercalated Na_*x*_CoO_2_^5^ and dome-shape relationship, similar to high temperature superconductor oxides, between transition temperature and doping is observed near *x* ≈ 0.30^6^. Also, large thermoelectric power was unveiled in Na_0.5_CoO_2_^7^. The Seebeck coefficient was strongly suppressed under magnetic field indicating the important role of spin-fluctuation^8^,^9^. All these properties originate from the archetype structure of hexagonal CoO_2_ layers which includes all the necessary ingredients for correlated electronic systems.

Inspired by the novel property of hexagonal CoO_2_ layers, recent studies have been extended to the so-called misfit layered Bi-A-Co-O compounds (A = Ca, Sr, Ba)^10^. Similar to Na_0.5_CoO_2_, a large thermoelectric power of over 100 *μ*V/K was observed in Bi_2_A_2_Co_2_O_8_ (A = Ca, Sr, Ba)^11^,^12^ and Ca_3_Co_4_O_9_^13^. Thus, the excellent thermoelectric property seems to be a common feature of hexagonal CoO_2_ layers. What is unique about misfit layered Bi_2_A_2_Co_2_O_8_ is an interesting insulator-metal (I-M) transition when either temperature or A’s ionic radius (*r*) increases^14^,^15^,^16^. Bi_2_Ca_2_Co_2_O_8_(BCCO) (*r*_*Ca*_ = 0.99 Å)^17^ and Bi_2_Sr_2_Co_2_O_8_ (BSCO)(*r*_*Sr*_ = 1.13 Å)^17^ with smaller *r* are insulators at low temperature and make a transition into metallic states at high temperature. The transition temperature *T*_*C*_ is around 140 K for Bi_2_Ca_2_Co_2_O_8_ and 60 K for Bi_2_Sr_2_Co_2_O_8_. Bi_2_Ba_2_Co_2_O_8_(BBCO) (*r*_*Ba*_ = 1.35 Å)^17^ with large *r* is different. It remains a metal and shows no insulator-metal transition as temperature varies^16^. Since high temperature means larger lattice constants of crystal structures, one is tempted to conclude that misfit layered Bi_2_A_2_Co_2_O_8_ is likely to take a metallic state at larger lattice constant while stays in an insulator state at smaller lattice constant.

To understand the nature of this insulator-metal transition, specific-heat data was very helpful. A sharp peak was observed to coincide with the transition temperature in Bi_2−*x*_Pb_*x*_Sr_2_Co_2_O_*z*_^14^. The insulator-metal transition is, thus, of the second order. Most strikingly, the magnitude of specific-heat peak strongly depends on external magnetic field and is suppressed when magnetic field is large (B = 13.2 T)^14^. The field-dependence of I-M transition is also confirmed by the zero-field-*μ*^+^-spin-rotation spectra where a magnetic anomaly is found at the transition temperature of BSCO^18^. Further implication involving spin-degree of freedom is the sizeable negative magnetoresistance observed in insulating Bi_2_Ca_2_Co_2_O_8_ and Bi_2_Sr_2_Co_2_O_8_ and positive magnetoresistance in metallic Bi_2_Ba_2_Co_2_O_8_^16^. In addition, the magnetic susceptibility of Bi_2_Ba_1.8_Co_2.2_O_8_ shows a typical Curie-law behavior near zero temperature and bump-like structure reminiscent to a nonmagnetic to magnetic transition at finite temperature^15^. However, the presence of Co_3_O_4_ clusters makes it difficult to identify the exact magnetic state involved in pure misfit layered compounds^19^. More recently, angle-resolved-photoemission spectra (ARPES) have been carried out for the electronic structure of Bi_2_Ba_2_Co_2_O_8_^20^,^21^,^22^. A hexagonal Fermi surface indicated that CoO_2_ layers are responsible for the metallic property^20^,^21^. Therefore, regarding the misfit layered Bi_2_A_2_Co_2_O_8_ compounds, important issues to be addressed are the natures of electronic states below and above the insulator-metal transition temperature.

The crystal structures of misfit layered Bi_2_A_2_Co_2_O_8_ are characterized by the so-called “composite structure” in which a CoO_2_ block and a rock-salt block composed of four layers of the types AO-BiO-BiO-AO interleave with each other^10^. The CoO_2_ block consists of a two-dimensional triangular lattice of Co ions, octahedrally coordinated with O ions above and below the Co plane. The formal valence of Co ions deduced from the chemical composition Bi_2_A_2_Co_2_O_8_ is 3+, leaving 6 electrons in Co-3*d* orbitals. In the edge sharing hexagonal CoO_2_ layer, the trigonal distortion of CoO_2_ octahedra reduces the *O*_*h*_ local point group to a *d*_3_ subgroup, further splitting the *t*_2*g*_ orbitals into an *a*_1*g*_ singlet and an 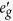 doublet^23^.

The previous local density approximation (LDA) study concentrated on the nonmagnetic state of Bi_2_A_2_Co_2_O_8_^16^. The Bi-*p* derived conduction bands and Co-*d*(*t*_2*g*_) derived valence bands overlap with each other and result in a metallic ground state. The metallic state explains the transport property of Bi_2_Ba_2_Co_2_O_8_ well, but is in contradiction with those of insulating Bi_2_Ca_2_Co_2_O_8_ and Bi_2_Sr_2_Co_2_O_8_. To reconcile the apparent discrepancy, Anderson weak localization mechanism was invoked and impurity-induced localization was attributed as a cause. If the Anderson weak localization mechanism is the sole cause for the insulator nature, one expects the negative magnetoresistance phenomena for all Bi_2_A_2_Co_2_O_8_ compounds. This is, however, not the case^16^. Also the suppression of the specific-heat peak and Seebeck coefficient under strong magnetic field cannot be understood since LDA calculation does not consider spin degrees of freedom. In fact, if Anderson weak localization mechanism was effective, carriers in Bi_2_Ba_2_Co_2_O_8_ would be the most vulnerable to localize since larger lattice constant implies narrower electronic bands. Important factors missing in previous theoretical calculation are strong electronic correlation and spin fluctuation. They are revealed by both the ARPES spectra^20^,^22^ and large thermoelectric power^9^. To manifest such effects, the Coulomb on-site repulsion on Co-*d* orbitals is an indispensable component. In fact, the on-site Coulomb repulsion modifies the above picture in two fundamental ways. Firstly, the Hubbard *U* pulls the occupied Co-*d*(*t*_2*g*_) orbitals downwards with respect to the unoccupied Bi-*p* and Co-*d*(*e*_*g*_) orbitals, thus a metal-insulator transition takes place eventually when *U* is large enough. This makes a correlation induced insulator possible^20^,^22^. Secondly, Hubbard *U* tends to forbid the double occupancy of Co-*d* orbitals, paving the way for magnetic moment formation.

In the prototype spin-state transition compound LaCoO_3_^24^, Co-*d* orbitals are decomposed into a *t*_2g_ triplet (*xy*, *yz*, *zx*) and *e*_*g*_ doublet (*x*^2^ − *y*^2^, 3*z*^2^ − *r*^2^) separated by a crystal-field-splitting 10*Dq*^25^,^26^. Depending on the competition between Hund’s rule coupling *J*_*H*_ and crystal-field-splitting energy 10*Dq*, low-spin (LS: 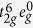, *S* = 0), intermediate-spin (IS: 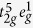, *S* = 1), and high-spin (HS: 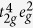, *S* = 2) states are all possible candidates^25^,^26^,^27^,^28^. In particular, 10*Dq* can easily be tuned either by ionic radius or temperature since it is inversely proportional to the fifth power of lattice constant. Previous studies showed that LaCoO_3_ experiences two physical transitions as temperature increases^24^. The first one takes place at 90 K and brings LaCoO_3_ from a nonmagnetic low-spin insulator to a paramagnetic insulator. The second one at 500 K eventually brings the compound into a paramagnetic metal. The high temperature phases are all connected with either IS state or HS state^27^,^28^.

Above analysis suggests that electronic structure and spin-state strongly depend on the exact A’s ionic radius and Coulomb repulsion *U* on Co-*d* orbitals. The insulator-metal transition in Bi_2_A_2_Co_2_O_8_ may have the same origin as that of the well-known spin-state transition in LaCoO_3_^25^,^26^. To seek for the most probable ground states of Bi_2_A_2_Co_2_O_8_ as a function of ionic radius, a comprehensive numerical calculation has been carried out in this paper within the local-spin density approximation plus *U* scheme (LSDA + *U*). In particular, the relative stabilities are sought for in (*U*, *r*) parameter space among different spin-states of fully relaxed crystal structures. Consistent with experimental observations, our study shows that only LS state and hexagonally arranged IS-LS mixed-state are the most probable candidates for ground states in the phase diagram. These two states offer consistent description of the physical properties of Bi_2_A_2_Co_2_O_8_ both below and above the transition temperature and the insulator-metal transition separating Bi_2_Ca_2_Co_2_O_8_, Bi_2_Sr_2_Co_2_O_8_, and Bi_2_Ba_2_Co_2_O_8_ compounds.

## Results

### Relaxed P-1 crystal structure of Bi_2_A_2_Co_2_O_8_

**Figure 1:**
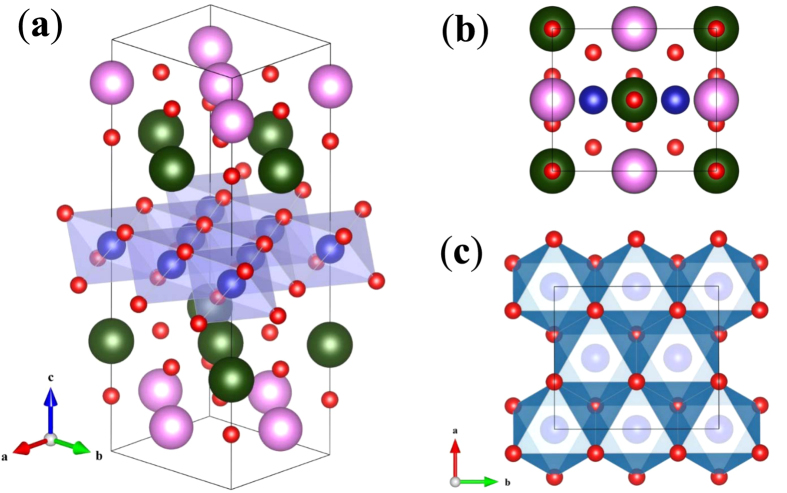
The crystal structure of Bi_2_A_2_Co_2_O_8_. The green (dark) and pink (light) large spheres refer to A(Ca, Sr, Ba) and Bi atoms, the blue (middle) and red (small) spheres refer to Co and O atoms. (**a**) Crystal structure. (**b**) The projected structure on *ab* plane. (**c**) The extracted hexagonal structure of CoO_2_ block viewed from *c*-axis.

An initial 28-atom unit cell of (Bi_2_A_2_O_4_)_2_(CoO_2_)_4_ is adopted to best describe the measured superstructure, in which the rectangular lattice of rock salt block (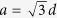, *b* = 2*d*) and hexagonal lattice of CoO_2_ block (*d*) form a commensurate periodic structure shown in Fig. 1. This structure is first optimized using local-density approximation (LDA) for the lattice parameters and atomic positions assuming the LS state of Co^3+^ ions. Then the crystal energy is further optimized by reducing crystal symmetry. P-1 crystal group is finally obtained and its optimized lattice parameters are listed in Table 1 together with those of measured ones^14^,^15^,^29^ listed in Table 2. The overall agreement is reasonable and discrepancy mainly results from the extra oxygens in the non-stoichiometric samples.

### Two types of competing ground states for Bi_2_A_2_Co_2_O_8_

The LDA electronic band structures of Bi_2_A_2_Co_2_O_8_ reproduced the metallic property obtained in previous study due to the overlap between Bi-*p* derived conduction bands and Co-*d*(*t*_2*g*_) derived valence bands. Although the result agrees with the metallic property of Bi_2_Ba_2_Co_2_O_8_, it disagrees with the insulator properties of Bi_2_Ca_2_Co_2_O_8_ and Bi_2_Sr_2_Co_2_O_8_. However, the band-overlap can be removed once Hubbard *U* increases to a critical value, and a correlation induced metal-insulator transition becomes possible^20^,^22^. The critical *U*_*C*_ is 5.80, 6.55, 6.25 eV for A = Ca, Sr, and Ba, respectively. The non-monotonic behavior of *U*_*C*_ is caused by the complex structural change as a function of A’s ionic radius. Since Hubbard *U* is intra-ionic in nature and only depends weakly on structural details, it is impossible to choose one value of *U* to fit the transport properties of all three Bi_2_A_2_Co_2_O_8_ compounds. Furthermore, LS state of Bi_2_A_2_Co_2_O_8_ does not include spin degree of freedom, so magnetic-field suppressed spin-fluctuation phenomena cannot be understood. This suggests that strong Hubbard *U* induced IS or HS state of Co ions must be a possibility.

**Figure 2:**
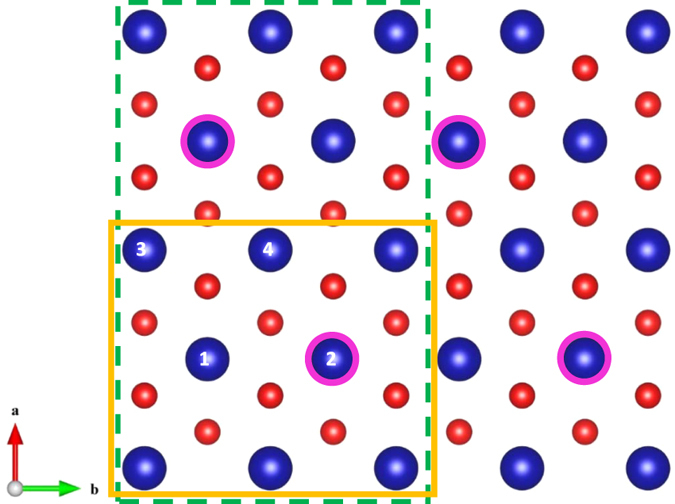
The sketch of projected hexagonal structure of CoO_2_ block on *ab* plane. The blue (big) spheres refer to Co ions while red (small) spheres refer to oxygen ions. The yellow (solid) rectangle frame denotes the unit cell used for LS state, the green (dashed) rectangle frame refers to the double-cell used for IS-LS mixed-state. The blue (big) spheres with red (shadowed) circles denote the IS Co ions of the IS-LS mixed-state.

As shown in Fig. 2, (Bi_2_A_2_O_4_)_2_(CoO_2_)_4_ unit cell contains four Co ions. Two of the Co ions (Co-3, Co-4) of CoO_6_ octahedra sit between the A and O atoms of AO rock-salt layers and their positions are equivalent. Other two (Co-1, Co-2) sit between the interstitial regions of rock-salt layers and are different. To find out the most probable spin structures of CoO_2_ block, we compare the energies of different spin-states of Co ions and spin structures with different combinations of LS, IS, and HS states in the unit cell. Our systematic study shows that uniform LS state is consistently lower in energy (0.416 eV at *U* = 8.0 eV) than the uniform IS state, IS state is much lower in energy (1.119 eV at *U* = 8.0 eV) than the uniform HS state. Thus, for uniform spin structures, LS state is the best candidate for the ground state of Bi_2_A_2_Co_2_O_8_. It remains to find out the other relevant magnetic structure. To this end, we first consider one, two, and three IS (HS) Co^3+^ ions in the background of LS Co^3+^ ions.

For single IS- or HS-Co^3+^ ions embedded in the background of LS-Co^3+^ ions in the unit cell, we have six inequivalent spin-structures. Table 3 shows the typical energies calculated at *U* = 8 eV. It is found that the energies of single IS-Co^3+^ cases are always lower than those of single HS-Co^3+^ cases, thus we focus on IS-state only below for more complex magnetic structures. In fact, single IS-Co^3+^-ion embedded in the background of LS-Co^3+^ ions can have even lower energy than that of the uniform LS state when *U* is larger while the opposite is true when *U* is smaller. To check whether single IS-Co^3+^-ion embedded in the background of LS-Co^3+^ ions is the most favored magnetic state, we have also considered double and triple IS-Co^3+^-ions embedded in LS-Co^3+^ ions and their energies are listed in Tables 4, 5 and 6. From Table 4 for parallel-spin and Table 5 for anitiparallel-spin configurations, double IS-Co^3+^-ions at Co-1 and Co-2 positions with parallel spins yields the lowest energy among the classes, but the energy is higher by 161.1 meV than that of single IS-Co^3+^-ion at Co-1. Also, triple IS-Co^3+^-ions case is higher in energy by 463.6 meV than that of single IS-Co^3+^-ion at Co-1. Thus, we conclude that single IS-Co^3+^-ion at Co-1 position has the lowest energy for one unit-cell configuration.

To restore the hexagonal symmetry of the Fermi surface observed experimentally, we double the unit cell along *a*-axis. Because the single IS Co-1 and Co-2 configurations are very close in energy (≈2.8 meV at *U* = 8.0 eV), we interchange the spin states of Co-1 and Co-2 ions of the second unit cell. In this way, we arrived at the hexagonally arranged IS-LS mixed-state whose energy is further lowered by 52 meV (at *U* = 8.0 eV) per unit cell. We shall show that this state possesses all the essential properties of metallic state observed either in the high temperature phase of Bi_2_Ca_2_Co_2_O_8_ and Bi_2_Sr_2_Co_2_O_8_ or Bi_2_Ba_2_Co_2_O_8_ at full temperature range.

### Insulator-metal phase diagram of Bi_2_A_2_Co_2_O_8_

**Figure 3:**
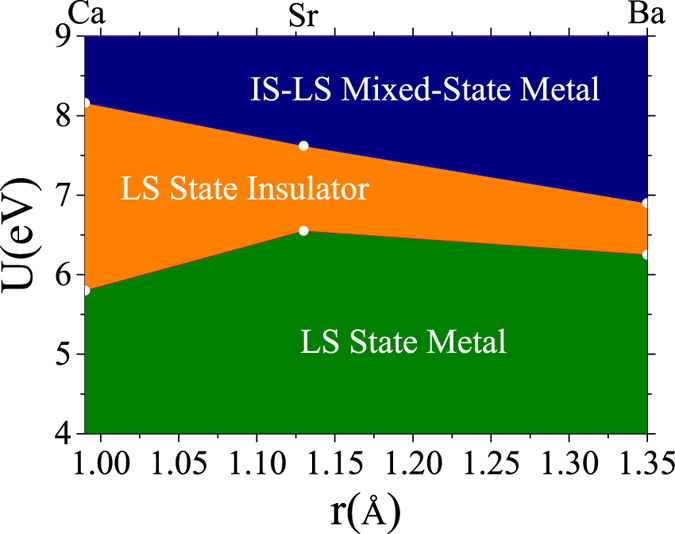
The phase diagram of insulator-metal transition of Bi_2_A_2_Co_2_O_8_ in (*U*, *r*) phase space. White spheres are the calculated critical *U*_*C*_ for A = Ca, Sr, Ba, the straight lines between the white spheres are drawn to guide eyes.

Although the hexagonally arranged IS-LS mixed-state is the most favored state in energy among the magnetic structures investigated, the relative stability between this state and uniform LS state depends on both the Hubbard *U* and A’s ionic radius. To quantitatively analyze the insulator-metal transition in Bi_2_A_2_Co_2_O_8_, we have numerically computed the phase diagram of these two electronic states in Fig. 3 using the same double-cell. The critical *U*_*C*_ separating the insulator (LS state) and metal (hexagonally arranged IS-LS mixed-state) is 8.16, 7,62, and 6.90 eV for Bi_2_Ca_2_Co_2_O_8_, Bi_2_Sr_2_Co_2_O_8_, and Bi_2_Ba_2_Co_2_O_8_, respectively. The monotonic decreasing value of *U*_*C*_ is directly related to the increased lattice constant, thus reduced crystal-field-splitting energy. The weakened crystal-field-splitting makes the IS-LS mixed-state possible. To conform to the experimentally observed transport properties of Bi_2_A_2_Co_2_O_8_ compounds, strong electronic correlation (6.90 eV < *U* < 7.62 eV) is required.

The Hubbard *U* and Hund’s exchange coupling *J*_*H*_ are usually extracted from fitting the core-electron satellite spectra of transition-metal oxides to a cluster model, and the values vary depending on the crystal structures and the valence of Co-ions. The values, 5 eV < *U* < 8 eV, *J*_*H*_ = 1 eV, recommended by Singh^23^ were widely cited for cobalt based perovskite. Korotin *et al*.^30^ obtained similar values (*U* = 7.8 eV, *J*_*H*_ = 0.92 eV) in discussing the IS state associated with the high temperature magnetic structure of LaCoO_3_. However, Solovyev, Hamada, and Terakura^31^ showed that effective Hubbard *U* of Co-*d*-orbitals depends on how to differentiate the localized orbitals from extended orbitals. They suggested *U* = 9.5eV if treating all *d*-orbitals as localized or a significantly smaller 2 eV for Co-*d*(*t*_2*g*_) orbitals if treating *d*(*t*_2*g*_) orbitals as localized while letting *d*(*e*_*g*_) orbitals extended. Thus, the value of Hubbard *U* is rather scattered, and this is the reason why we treat the Hubbard *U* as a parameter to derive the insulator-metal phase diagram. Our required Hubbard *U* (6.90 eV < *U* < 7.62 eV) is, however, within the accepted range.

### Electronic structures and atom-resolved partial densities of states

**Figure 4:**
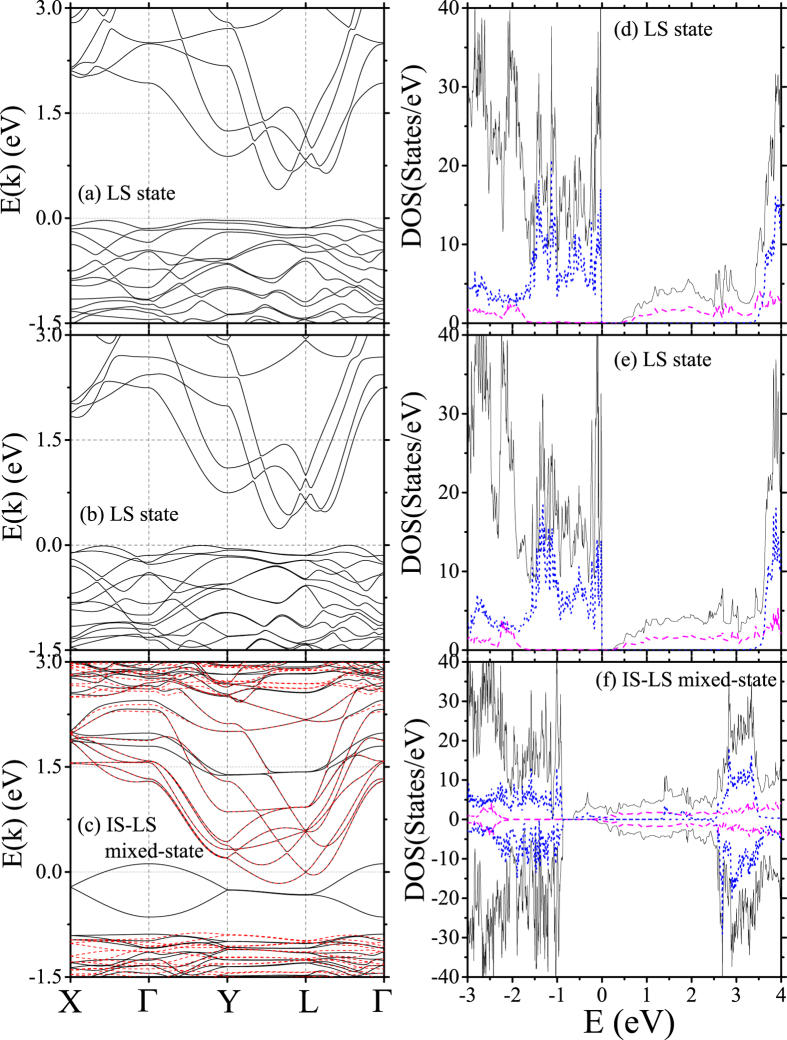
The band structures and densities of states of Bi_2_A_2_Co_2_O_8_ compounds. The black (solid) and red (dashed) lines in (**a**–**c**) refer to the bands for spin-up and spin-down electrons. The black (solid), blue (dotted), and pink (dashed) lines in (**d**–**f**) refer to the total, Co-, and Bi-resolved partial densities of states. The Fermi energy is set as 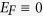 eV. (**a**,**d**) Bi_2_Ca_2_Co_2_O_8_; (**b**,**e**) Bi_2_Sr_2_Co_2_O_8_; (**c**,**f**) Bi_2_Ba_2_Co_2_O_8_.

To check the transport properties of Bi_2_A_2_Co_2_O_8_ compounds, the electronic band structures and atom-resolved partial densities of states (DOS) are calculated at *U* = 7.5 eV. They are presented in Fig. 4 for the LS state insulators of Bi_2_Ca_2_Co_2_O_8_ and Bi_2_Sr_2_Co_2_O_8_, and the IS-LS mixed-state metal of Bi_2_Ba_2_Co_2_O_8_, respectively. Left panel plots the band structures computed along the high symmetrical points X( 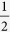
00), Γ(000), Y(0
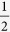
0), L(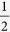

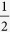
0), Γ(000) in the irreducible Brillouin zone, and right panel plots the total as well as Co and Bi atom-resolved partial densities of states. It is seen that Bi_2_Ca_2_Co_2_O_8_ and Bi_2_Sr_2_Co_2_O_8_ are indirect band-gap insulators, and the band-gaps are 0.416 eV and 0.236 eV, respectively. The valence bands derive from CoO_2_ layers with Co-*d*(*t*_2*g*_) character while conduction bands mainly come from BiO rock-salt layer. Bi_2_Ba_2_Co_2_O_8_ is a typical spin-polarized semi-metal due to the overlap between the spin-degenerated conduction bands derived from BiO layer and up-spin valence band from the *d*(*e*_*g*_) orbitals of CoO_2_ layer. The charge carriers involve both electrons and holes and exact charge carrier type depends on the stoichiometry of the compound.

The band characters mentioned above can be seen clearly from the corresponding atom-resolved densities of states shown in the right panel. While the valence bands originate from CoO_2_ layers and conduction bands originate from BiO layers for both the correlation-induced LS state insulator and hexagonally arranged IS-LS mixed-state metal, the orbital characters of valence bands show a notable difference for these two electronic states. For LS state insulator, all Co-spins are almost the same and local symmetry is high around Co ions. Co-*d* orbitals are splitted into a *d*(*t*_2*g*_) triplet and *d*(*e*_*g*_) doublet separated by about 3.5 eV. *d*(*t*_2*g*_) triplet are fully occupied and the nonmagnetic valence band is of Co-*d*(*t*_2*g*_) character. However, for IS-LS mixed-state metal, the presence of two types of Co-spins reduces the local symmetries both around LS and IS Co ions. In particular, Jahn-Teller distortion of IS CoO_6_ octahedra induces a bond elongation of 9% along *a*-axis which is significant enough to pull one of up-spin *e*_*g*_ orbitals down below the Fermi energy while push one of the down-spin *t*_2*g*_ orbitals high above the Fermi energy. This forms an IS-state of Co ions and valence band is of Co-*d*(*e*_*g*_) character. The magnetic moment of IS Co ions can be calculated from the Co-resolved partial DOS. This yields a value of 1.90 *μ*_*B*_ which is consistent with that of IS Co ions. For the detailed information on the spin states and valence states of Co-ions, the density matrices of Co-*d* orbitals are included in Supplementary Information. The diagonalized density matrices clearly show that all Co-ions of Bi_2_Ca_2_Co_2_O_8_ and Bi_2_Sr_2_Co_2_O_8_ are indeed in LS state while quarter of Co-ions of Bi_2_Ba_2_Co_2_O_8_ are in IS state. The valence states of Co-ions are somewhat less than Co^3+^ because of strong covalence bonding with oxygen atoms. We speculate that the Co^4+^-ions observed in the experiment^16^ are most probably due to the extra oxygen atoms presented in nonstoichiometric samples.

## Discussion

Now we are in position to discuss the various experimental phenomena with the above two electronic states. (1) LS state insulator and IS-LS mixed-state metal agree perfectly well with the insulating properties of Bi_2_Ca_2_Co_2_O_8_ and Bi_2_Sr_2_Co_2_O_8_ and the metallic property of Bi_2_Ba_2_Co_2_O_8_. If one associates the LS state insulator (stable at small A’s ionic radius) with low-temperature phase and IS-LS mixed-state metal (stable at large A’s ionic radius) with high-temperature phase as customarily done in spin-state transition phenomena^30^, the insulator-metal phase transition of Bi_2_Ca_2_Co_2_O_8_ and Bi_2_Sr_2_Co_2_O_8_ at finite temperatures can be also qualitatively understood with these two states. (2) The magnetic nature of the IS-LS mixed-state suggests that spin fluctuation is involved in the insulator-metal phase transition of Bi_2_Sr_2_Co_2_O_8_, thus the specific-heat peak caused by spin-fluctuation should be suppressed by external magnetic field as was observed in experiment^14^. (3) Since the ground states of stoichiometric Bi_2_A_2_Co_2_O_8_ (A = Ca, Sr) are nonmagnetic insulators, the electron and hole concentrations at low temperature are very diluted. The scattering probability is low and phase coherence is maintained. Thus Anderson weak localization mechanism works and back-scattering is dominant^32^. Applying external magnetic field destroys the back-scattering and results in a negative magnetoresistance^33^. For metallic Bi_2_Ba_2_Co_2_O_8_, finite carrier density is present at Fermi energy and scattering is strong. Electric conductivity is of diffusive type and positive magnetoresistance prevails. The linear B-dependent magnetoresistance may be related to the layered semi-metal property of Bi_2_Ba_2_Co_2_O_8_^34^. (4) Our LSDA + *U* band structure predicts a spin-polarized semi-metal for Bi_2_Ba_2_Co_2_O_8_, thus the transport and magnetoresistance phenomena can be qualitatively understood. But the LSDA + *U* band structures did not take the charge and spin fluctuations into account, direct comparison with the ARPES spectra is not feasible. To better describe the renormalized quasiparticle spectra, the dynamical mean-field theory for a supercell with 8 Co-ions (40 *d*-orbitals) has to be implemented which is a challenging issue. In addition, our study suggests that sizeable Hubbard *U* is required to stabilize both the insulator property of LS state and metallic property of IS-LS mixed-state. The strong electronic correlation is a prerequisite in explaining the ARPES spectra^20^,^22^ and thermoelectric properties of Bi_2_A_2_Co_2_O_8_ compounds^11^,^12^.

In summary, we propose in this paper that the LS state insulator and IS-LS mixed-state metal are the two electronic states involved in the insulator-metal transition observed in Bi_2_A_2_Co_2_O_8_ compounds. The physical properties of the two states offer satisfactory explanations not only to the magnetic-field suppressed specific-heat peak associated with the insulator-metal transition, but also to the distinct magnetoresistance features observed in Bi_2_Ca_2_Co_2_O_8_ and Bi_2_Sr_2_Co_2_O_8_, and Bi_2_Ba_2_Co_2_O_8_. Although the conclusion is mainly drawn based on Bi_2_A_2_Co_2_O_8_ series of compounds, it is of interest to explore whether similar phenomena appear in other hexagonally structured CoO_2_ compounds.

## Methods

### The parameter setting for density functional theory

To search for the relevant electronic states, the calculations were performed by using a plane wave pseudopotential approach to the density-functional theory (DFT) as implemented in the Vienna *ab initio* Simulation Package (VASP 5.3.5)^35^,^36^. We use the Ceperley-Alder (CA) functional as the exchange-correlation potential^37^. The projector augmented wave (PAW) potentials explicitly include 10 valence electrons for Ca (Sr, Ba) (3*s*^2^3*p*^6^4*s*^2^), 17 for Co (3*s*^2^3*p*^6^3*d*^7^4*s*^2^), 5 for Bi (6*s*^2^6*p*^3^) and 6 for O (2*s*^2^2*p*^4^). The default PAW spheres sizes are supplied by VASP program. The rotationally invariant LSDA + *U* approach introduced by Liechtenstein *et al*.^38^ was adopted for Co 3*d* orbitals. The wave functions are expanded using a plane-waves basis with an energy cutoff of 600 eV. 8 × 8 × 2 and 4 × 8 × 2 Monkhorst-Pack *k*-point meshes are used for (Bi_2_A_2_O_4_)_2_(CoO_2_)_4_ unit cell (28 atoms) and double-cell, respectively^39^. The total energy and density of states were calculated using the linear tetrahedron method with Bloch corrections^40^. Each self-consistent electronic calculation is converged to 10^−5^ eV and the tolerance force is set to 0.01 eV/Å for ionic relaxation. As was usually done for later transition-metal element Co, the Hund’s rule coupling *J*_*H*_ was fixed at 1 eV^31^ while on-site Hubbard *U* is allowed to vary between 5 to 8 eV according to Singh^23^. To obtain the self-consistent electronic structures and total-energies, we first set up an initial spin configuration of Co-ions according to the specific spin-states. The orbital and spin electron occupations are then allowed to relax and are iterated until full convergence. The converged spin-states of Co-ions are then determined by the integrated magnetic moments. In this way, we are able to map the phase diagram of misfit layered Bi_2_A_2_Co_2_O_8_ compounds with different *U* and A’s ionic radius.

## Additional Information

**How to cite this article**: Huang, X. and Zhang, W. How does the spin-state of Co ions affect the insulator-metal transition in Bi_2_A_2_Co_2_O_8_ (A=Ca, Sr, Ba)? *Sci. Rep.* **6**, 38212; doi: 10.1038/srep38212 (2016).

**Publisher’s note:** Springer Nature remains neutral with regard to jurisdictional claims in published maps and institutional affiliations.

## Supplementary Material

Supplementary Information

## Figures and Tables

**Table 1:** The lattice parameters of calculated P-1 crystal structure of Bi_2_A_2_Co_2_O_8_.

	*a*	*b*	*c*	*α*	*β*	*γ*
Ca	4.66	5.49	14.32	90.40	94.10	90.32
Sr	4.77	5.47	14.72	90.10	94.26	89.97
Ba	4.89	5.53	15.23	89.95	94.11	89.89

The lattice constants are in unit of Å and angles are in unit of degree.

**Table 2:** The lattice parameters of measured misfit layered compounds.

	*a*	*b*	*c*	*3*	*β*	*γ*
Bi_2_Ca_2_Co_*x*_O_8+*δ*_	—	—	14.65	—	—	—
Bi_2_Sr_2_Co_1.87_O_8+*δ*_	4.94	5.39	14.96	—	93.5	—
Bi_2_Ba_1.8_Co_2.2_O_8+*δ*_	4.884	5.639	15.43	—	—	—

The lattice constants are in unit of Å and angles are in unit of degree.

**Table 3:** The total energy per unit cell of one IS (HS)-Co^3+^ ion embedded in the background of LS-Co^3+^-ions of Bi_2_A_2_Co_2_O_8_.

Type of spin-state	Total energy E (eV)	E-E (LS) (meV)
IS(Co-1)	−179.6432	−203.6
IS(Co-2)	−179.6404	−200.8
IS(Co-3,Co-4)	−179.5889	−149.3
HS(Co-1)	−179.3828	56.8
HS(Co-2)	−179.3751	64.5
HS(Co-3,Co-4)	−179.3019	137.7

(28 atoms).

**Table 4:** The total energy per unit cell of two IS-Co^3+^ ions embedded in the background of LS-Co^3+^ ions of Bi_2_A_2_Co_2_O_8_.

Type of spin-state	Total energy E (eV)	E-E (LS) (meV)
IS-Co-1, IS-Co-2	−179.4821	−42.5
IS-Co-1, IS-Co-3 or IS-Co-4	−179.3088	130.8
IS-Co-2, IS-Co-3 or IS-Co-4	−179.3072	132.4
IS-Co-3, IS-Co-4	−179.4449	−5.3

(28 atoms, IS parallel).

**Table 5:** The total energy per unit cell of two IS-Co^3+^ ions embedded in the background of LS-Co^3+^ ions of Bi_2_A_2_Co_2_O_8_.

Type of spin-state	Total energy E (eV)	E-E (LS) (meV)
IS-Co-1, IS-Co-2	−179.4312	8.4
IS-Co-1, IS-Co-3 or IS-Co-4	−179.3627	76.9
IS-Co-2, IS-Co-3 or IS-Co-4	−179.3619	77.7
IS-Co-3, IS-Co-4	−179.4242	15.4

(28 atoms, IS antiparallel).

**Table 6:** The total energy per unit cell of three parallel IS-Co^3+^ ions embedded in the background of LS-Co^3+^ ions of Bi_2_A_2_Co_2_O_8_.

Type of spin-state	Total energy E (eV)	E-E (LS) (meV)
LS-Co-1	−179.1029	336.7
LS-Co-2	−179.0974	342.2
LS-Co-3 or LS-Co-4	−179.1796	260.0

(28 atoms).
