# Supplementary material for: How does the spin-state of Co ions affect the insulator-metal transition in Bi2A2Co2O8 (A = Ca, Sr, Ba)?
Source: Sci Rep. 2016 Nov 30;6:38212. doi: 10.1038/srep38212 (PMC5128795; doi:10.1038/srep38212)
Supplement: Supplementary Information [file srep38212-s1.pdf]

Supplementary Information for "How does the spin-state of Co ions affect the insulator-metal transition in $Bi_2A_2Co_2O_8$ (A=Ca, Sr, Ba)?"

Xiaokun Huang & Weiyi Zhang*

National Laboratory of Solid State Microstructures and Department of Physics, Nanjing University, Nanjing 210093, China

Collaborative Innovation Center of Advanced Microstructures, Nanjing University, Nanjing 210093, China

*Corresponding author: wyzhang@nju.edu.cn

This Supplementary Information includes following parts:

(1) The convergence checks on k-points samplings for the total energy of LS-state of $Bi_2Ba_2Co_2O_8$. (U=8 eV, J_H=1eV)

(2) The relative stabilities among different spin structures of $Bi_2Ba_2Co_2O_8$ (the yellow line framed unit cell, 28 atoms) calculated at U=8 eV, J_H =1eV, and $8 \times 8 \times 2$ k-points sampling.

(3) The hexagonally arranged IS-LS mixed-state of $Bi_2Ba_2Co_2O_8$ (the green line framed double-cell, 56 atoms) calculated at U=8 eV, J_H=1eV, and $4\times 8 \times 2$ k-points sampling.

(4) The characteristic band-structures and densities of states of $Bi_2A_2Co_2O_8$ (A=Ca, Sr, Ba) for the LS state metal (U=4.5 eV), LS state insulator (U=6.8 eV), and IS-LS mixed-state metal (U=8.3 eV).

(5) The projected Co-d-orbitals occupations of $Bi_2Ca(Sr)_2Co_2O_8$ in LS state and $Bi_2Ba_2Co_2O_8$ in hexagonally arranged IS-LS mixed-state at U=7.5 eV and J_H=1eV.

(1) The convergence checks on k-points samplings for the total energy of LS-state of $Bi_2Ba_2Co_2O_8$. (U=8 eV，J_H=1eV)

In the total energy calculations we use 8×8×2 and 4×8×2 Γ-centered *k*-points samplings for the unit cell (28 atoms) and double-cell (56 atoms), and the energy convergence checks are displayed in Table S1 and Table S2. An absolute error in energy is less than 0.1meV per unit cell using the parameter settings above. Therefore, excellent numerical convergences have been achieved on total energies.

Table S1. The total energy per unit cell of LS-state of $Bi_2Ba_2Co_2O_8$. (28 atoms)

K mesh	E (eV)
$6 \times 6 \times 2$	-179.439613
$8 \times 8 \times 2$	-179.439630
$8 \times 8 \times 4$	-179.439699
$10 \times 10 \times 2$	-179.439661

Table S2. The total energy for double-cell of LS-state of $Bi_2Ba_2Co_2O_8$. (56 atoms)

K mesh	E (eV)
$4 \times 6 \times 2$	-358.880598
$4 \times 8 \times 2$	-358.880736
$6 \times 8 \times 2$	-358.880699
$4 \times 10 \times 2$	-358.880762
$6 \times 10 \times 2$	-358.880517

(2) The relative stabilities among different spin structures of $Bi_2Ba_2Co_2O_8$ (the yellow line framed unit cell, 28 atoms) calculated at U=8 eV, J_H=1eV, and $8 \times 8 \times 2$ k-points sampling.

Co^{3+} ions have three different spin states, i.e. low-spin state ($t_{2g}^6 e_g^0$, S=0, LS), intermediate-spin state ($t_{2g}^5 e_g^1$, S=1, IS), and high-spin state ($t_{2g}^4 e_g^2$, S=2, HS). To find out the best candidates for the two electronic states below and above the insulator-metal

transition temperature, we search for the possible ground states of the compounds (unit cell of Figure S1) in following steps. (1) The uniform spin-states of the four Co^{3+} ions in unit cell: As shown in Table S3, the uniform LS-state has the lowest energy among uniform LS-, IS-, and HS-states. (2) Single IS- or HS-Co^{3+} ions embedded in the background of LS-Co^{3+} ions in unit cell: As Co-3 and Co-4 positions are equivalent in the P-1 crystal group (see Figure S1), we only consider six spin structures, i.e. single IS- or HS-Co^{3+} at Co-1, Co-2, or Co-3 positions. The results are summarized in Table S4. It is seen that the energies of single IS-Co^{3+} cases are always lower than those of single HS-Co^{3+} cases, thus we focus on IS-state only below for more complex magnetic structures. In fact, single IS-Co^{3+}-ion embedded in the background of LS-Co^{3+} ions can have even lower energy than that of the uniform LS-state when U is larger while the opposite is true when U is smaller. To check whether single IS-Co^{3+}-ion embedded in the background of LS-Co^{3+} ions is the most favored magnetic state, we have also considered double and triple IS-Co^{3+}-ions embedded in LS-Co^{3+} ions and their energies are listed in Tables S5-S7. From Table S5 and Table S6, double IS-Co^{3+}-ion at Co-1 and Co-2 positions with parallel spins yields the lowest energy among its class, but the energy is higher by 161.1meV than that of single IS-Co^{3+}-ion at Co-1. Also, triple IS-Co^{3+}-ion case is higher in energy by 463.6meV than that of single IS-Co^{3+}-ion at Co-1. Thus, we conclude that single IS-Co^{3+}-ion at Co-1 position has the lowest energy for one unit-cell configuration.

Table S3. The total energy per unit cell of uniform spin-states of $Bi_2Ba_2Co_2O_8$. (28 atoms)

Type of spin-state	Total energy E (eV)	E-E(LS) (meV)
LS(S=0)	-179.4396	0

IS(S=1)	-179.0236	416.0
HS(S=2)	-177.9051	1534.5

Table S4. The total energy per unit cell of one IS (HS)-Co^{3+} ion embedded in the background of LS-Co^{3+}-ions of $Bi_2Ba_2Co_2O_8$. (28 atoms)

Type of spin structure	Total energy E (eV)	E-E(LS) (meV)
IS(Co-1)	-179.6432	-203.6
IS(Co-2)	-179.6404	-200.8
IS(Co-3,Co-4)	-179.5889	-149.3
HS(Co-1)	-179.3828	56.8
HS(Co-2)	-179.3751	64.5
HS(Co-3,Co-4)	-179.3019	137.7

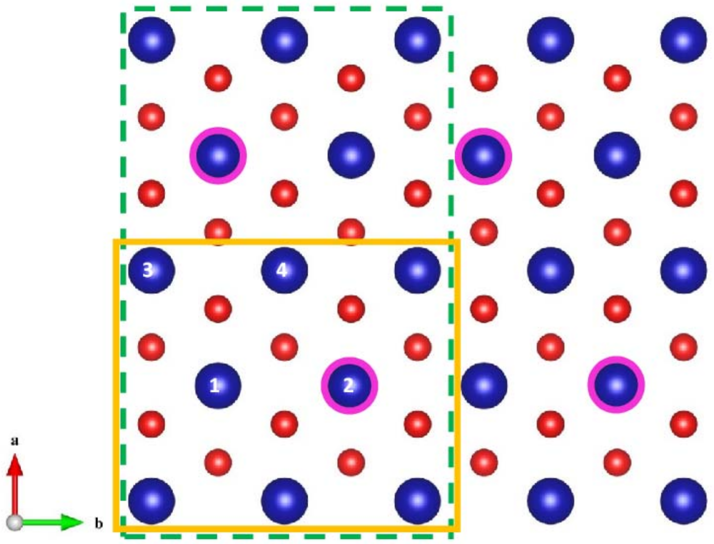

Figure S1 The sketch of projected hexagonal structure of CoO_2 block on *ab* plane. The blue (big) spheres refer to Co ions while red (small) spheres refer to oxygen ions. The yellow (solid) rectangle frame denotes the unit cell used for LS-state, the green (dashed) rectangle frame refers to the double-cell used for IS-LS mixed-state. The blue (big) spheres with red (shadowed) shells denote the IS-state Co ions of the IS-LS mixed-state.

Table S5. The total energy per unit cell of two IS-Co^{3+} ions embedded in the background of LS-Co^{3+} ions of $Bi_2Ba_2Co_2O_8$. (28 atoms, IS parallel)

Type of spin structure	Total energy E (eV)	E-E (LS)(meV)
IS-Co-1, IS-Co-2	-179.4821	-42.5
IS-Co-1, IS-Co-3 or IS-Co-4	-179.3088	130.8
IS-Co-2, IS-Co-3 or IS-Co-4	-179.3072	132.4
IS-Co-3, IS-Co-4	-179.4449	-5.3

Table S6. The total energy per unit cell of two IS-Co^{3+} ions embedded in the background of LS-Co^{3+} ions of $Bi_2Ba_2Co_2O_8$. (28 atoms, IS antiparallel)

Type of spin structure	Total energy E (eV)	E-E (LS)(meV)
IS-Co-1, IS-Co-2	-179.4312	8.4
IS-Co-1, IS-Co-3 or IS-Co-4	-179.3627	76.9
IS-Co-2, IS-Co-3 or IS-Co-4	-179.3619	77.7
IS-Co-3, IS-Co-4	-179.4242	15.4

Table S7. The total energy per unit cell of three parallel IS-Co^{3+} ions embedded in the background of LS-Co^{3+} ions of $Bi_2Ba_2Co_2O_8$. (28 atoms)

Type of spin structure	Total energy E (eV)	E-E (LS)(meV)
LS-Co-1	-179.1029	336.7
LS-Co-2	-179.0974	342.2
LS-Co-3 or LS-Co-4	-179.1796	260.0

(3) The hexagonally arranged IS-LS mixed-state of $Bi_2Ba_2Co_2O_8$ (the green line framed double-cell, 56 atoms) calculated at U=8 eV, J_H=1eV, and $4\times 8\times 2$ k-points sampling.

To restore the hexagonal symmetry of the Fermi surface observed experimentally, we double the unit cell along *a*-axis. Because the single IS Co-1 and Co-2 configurations are very close in energy (2.8 meV at U=8.0 eV), we interchange the spin states of Co-1 and Co-2 ions of the second unit cell (see Figure S2). In this way, we arrived at the hexagonally arranged IS-LS mixed-state whose energy is further lowered by 52 meV

(at U=8.0 eV) per unit cell. Another possible hexagonally arranged IS-LS mixed-state is depicted in Figure S3 with IS-Co^{3+} takes the Co-3 position of the first unit cell and Co-4 position in the second unit-cell. However, this state is higher in energy by 95.6meV per unit cell than the Co-1 Co-2 configuration. Thus, we conclude that Co-1 Co-2 hexagonally arranged IS-LS mixed state is the spin-state competing with the uniform LS-state.

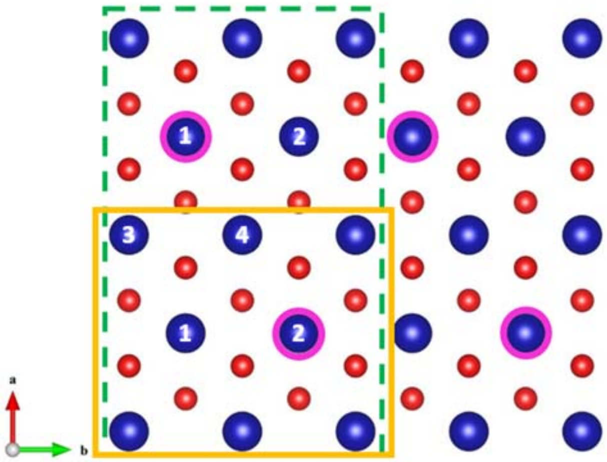

Figure S2 The hexagonally arranged IS-LS mixed-state with IS-Co^{3+} ions at Co-1 and Co-2 positions.

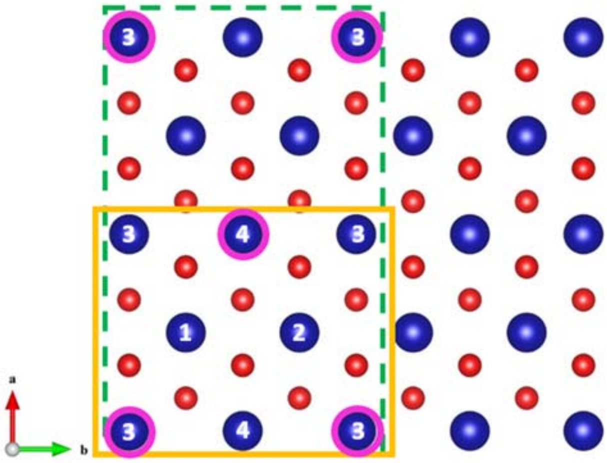

Figure S3 The hexagonally arranged IS-LS mixed-state with IS-Co^{3+} ions at Co-3 and Co-4 positions.

Table S8. The hexagonally arranged IS-LS mixed-state of $Bi_2Ba_2Co_2O_8$ (the green line framed double-cell, 56 atoms) calculated at U=8 eV, J_H=1eV, and 4× 8 × 2 k-points sampling.

Type of spin structure	Total energy E(eV) per double-cell	Total energy E (eV) per unit-cell	E-E (LS)(meV) per unit-cell
Co-1 and Co-2	-359.39107	-179.6956	-256.0
Co-3 and Co-4	-359.19985	-179.6000	-160.4

(4) The characteristic band-structures and densities of states of $Bi_2A_2Co_2O_8$ (A=Ca, Sr, Ba) for the LS state metal (U=4.5 eV), LS state insulator (U=6.8 eV), and IS-LS mixed-state metal (U=8.3 eV).

To illustrate the typical electronic spectra for the three phases studied in this paper, the LS state metal (marked green in Fig. 3, U=4.5 eV), LS state insulator (marked orange in Fig. 3, U=6.8 eV), and IS-LS mixed-state metal (marked blue in Fig. 3, U=8.3 eV) are shown in Figure S4-S6, respectively.

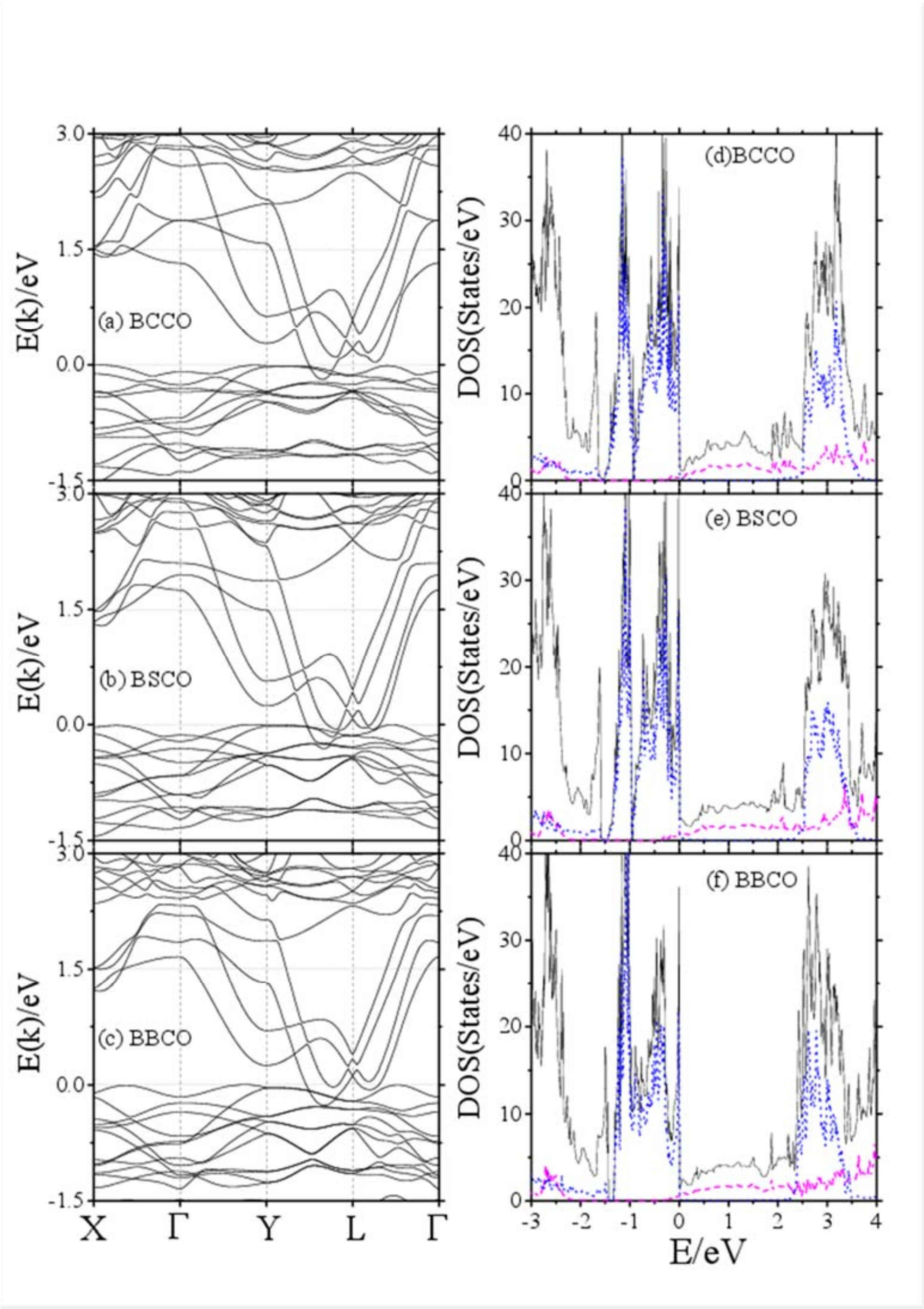

Figure S4 The typical band structures and densities of states of LS state metal of $Bi_2A_2Co_2O_8$ (A=Ca, Sr, Ba) obtained at U=4.5 eV. (**a**)-(**c**) refer to band structures with black and red lines denoting the spin-up and spin-down bands. (**d**)-(**f**) refer to densities of states with black (solid), blue (dotted), and pink (dashed) lines denoting the total, Co- and Bi-resolved partial densities of states. Fermi energy is set as E_F=0eV. (**a**)+(**d**) $Bi_2Ca_2Co_2O_8$; (**b**)+(**e**) $Bi_2Sr_2Co_2O_8$; (**c**)+(**f**) $Bi_2Ba_2Co_2O_8$.

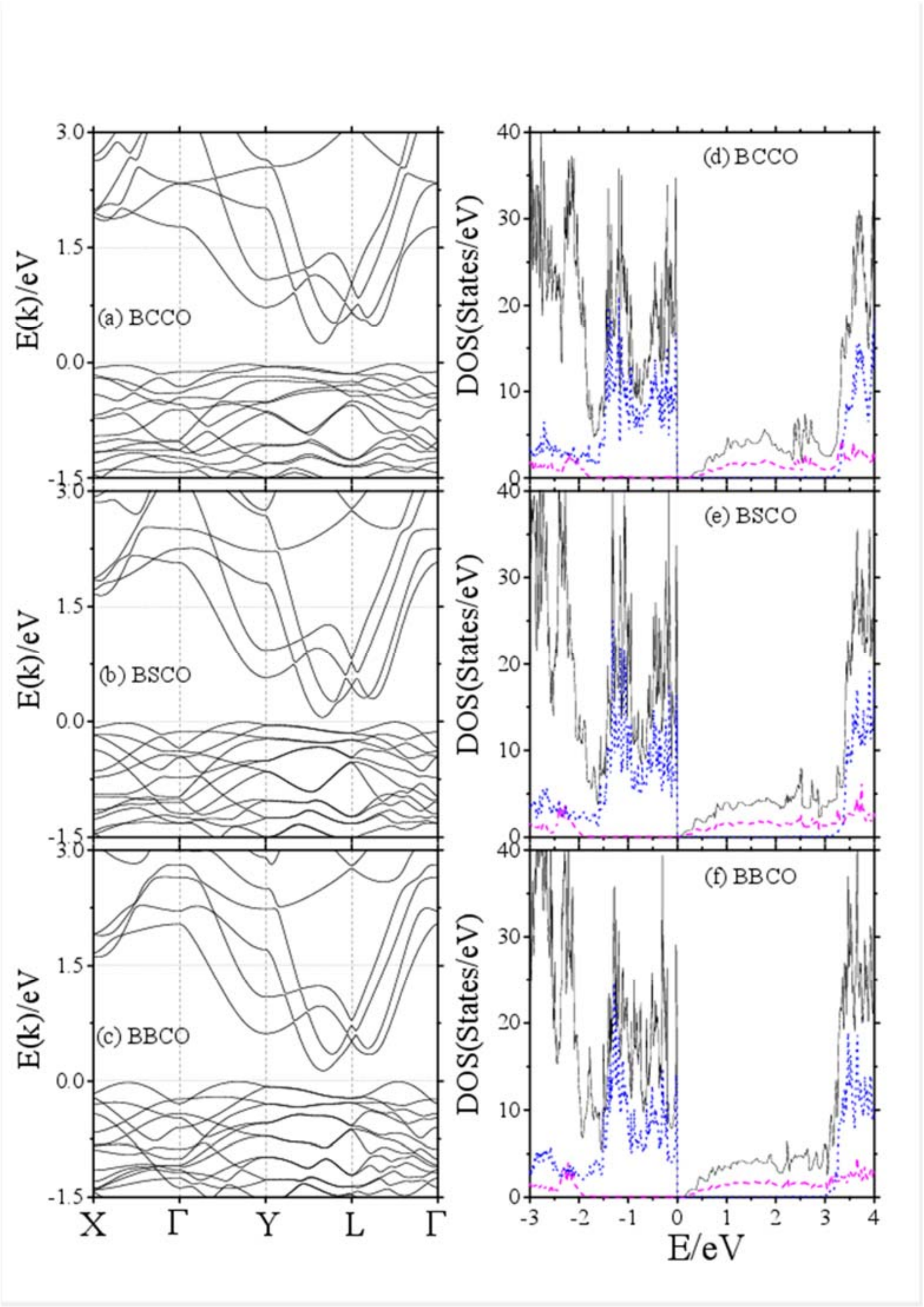

Figure S5 The typical band structures and densities of states of LS state insulator of $Bi_2A_2Co_2O_8$ (A=Ca, Sr, Ba) obtained at U=6.8 eV. (**a**)-(**c**) refer to band structures with black and red lines denoting the spin-up and spin-down bands. (**d**)-(**f**) refer to densities of states with black (solid), blue (dotted), and pink (dashed) lines denoting the total, Co- and Bi-resolved partial densities of states. Fermi energy is set as E_F=0eV. (**a**)+(**d**) $Bi_2Ca_2Co_2O_8$; (**b**)+(**e**) $Bi_2Sr_2Co_2O_8$; (**c**)+(**f**) $Bi_2Ba_2Co_2O_8$.

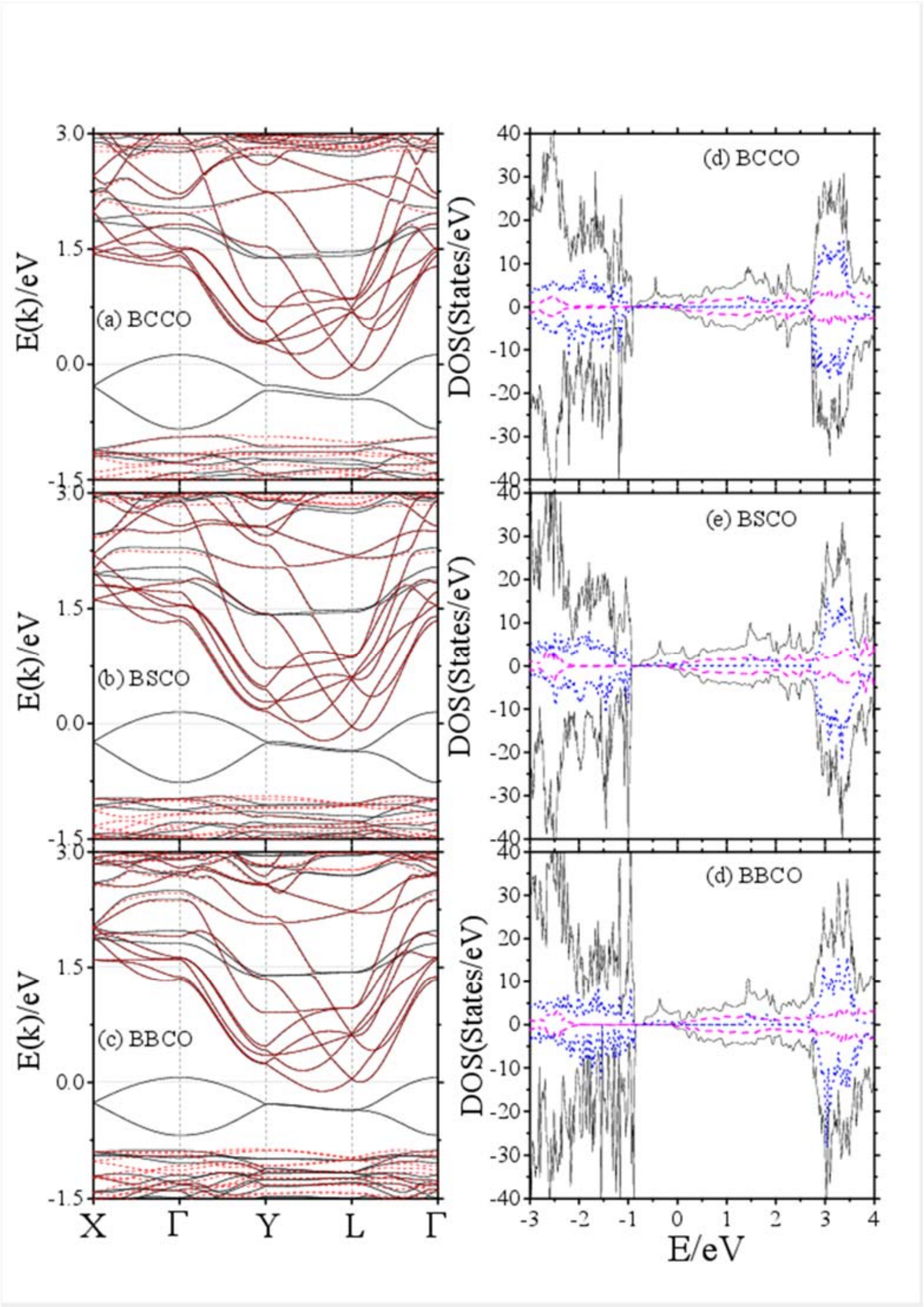

Figure S6 The typical band structures and densities of states of IS-LS mixed-state metal of $Bi_2A_2Co_2O_8$ (A=Ca, Sr, Ba) obtained at U=8.3 eV. (**a**)-(**c**) refer to band structures with black and red lines denoting the spin-up and spin-down bands. (**d**)-(**f**) refer to densities of states with black (solid), blue (dotted), and pink (dashed) lines denoting the total, Co- and Bi-resolved partial densities of states. Fermi energy is set as E_F=0eV. (**a**)+(**d**) $Bi_2Ca_2Co_2O_8$; (**b**)+(**e**) $Bi_2Sr_2Co_2O_8$; (**c**)+(**f**) $Bi_2Ba_2Co_2O_8$.

(5) The projected Co-d-orbitals occupations of $Bi_2Ca(Sr)_2Co_2O_8$ in LS state and $Bi_2Ba_2Co_2O_8$ in hexagonally arranged IS-LS mixed-state at U=7.5 eV and J_H=1eV.

In the following, the on-site Co-d density matrix M, its diagonalized matrix M_{dig} and associated eigenvector matrix M_{vec} are listed for each inequivalent Co sites. These three matrices are related by the following eigen-equation

$$M_{vec}^{\dagger} \, M \, M_{vec} = M_{dig}$$

The density matrix M is defined on the basis vectors in the order d_{xy} d_{yz} $d_{3z^2-r^2}$ d_{xz} $d_{x^2-y^2}$. Here x and y are taken along the crystal axes a, b. z is perpendicular to ab plane.

In LS-state, there are three inequivalent Co-ions in $Bi_2Ca(Sr)_2Co_2O_8$, they are Co-1, Co-2, and Co-3(Co-4).

The corresponding density matrices M, M_{dig}, M_{vec} are

Ca-Co-1-LS-up(down)

$$\begin{bmatrix} 0.7144 & -0.3025 & -0.0018 & 0.0107 & -0.0004 \\ -0.3025 & 0.5186 & -0.0027 & 0.0023 & 0.0078 \\ -0.0018 & -0.0027 & 0.9336 & 0.0203 & -0.0129 \\ 0.0107 & 0.0023 & 0.0203 & 0.4948 & 0.3018 \\ -0.0004 & 0.0078 & -0.0129 & 0.3018 & 0.7261 \end{bmatrix}$$

$$\begin{bmatrix} 0.2993 & 0 & 0 & 0 & 0 \\ 0 & 0.2854 & 0 & 0 & 0 \\ 0 & 0 & 0.9354 & 0 & 0 \\ 0 & 0 & 0 & 0.9343 & 0 \\ 0 & 0 & 0 & 0 & 0.9332 \end{bmatrix}$$

$$\begin{bmatrix} 0.5663 & -0.1595 & -0.4181 & -0.6766 & 0.1462 \\ 0.7843 & -0.1968 & 0.3006 & 0.4980 & -0.0879 \\ -0.0046 & -0.0371 & -0.7423 & 0.3389 & -0.5769 \\ 0.2001 & 0.7987 & -0.2707 & 0.2409 & 0.4368 \\ -0.1554 & -0.5446 & -0.3325 & 0.3483 & 0.6688 \end{bmatrix}$$

Ca-Co-2-LS-up(down)

$$\begin{bmatrix} 0.7170 & -0.2991 & 0.0096 & -0.0180 & 0.0118 \\ -0.2991 & 0.5210 & 0.0136 & 0..0094 & -0.0081 \\ 0.0096 & 0.0136 & 0.9324 & 0.0146 & -0.0090 \\ -0.0180 & 0.0094 & 0.0146 & 0.4995 & 0.3046 \\ 0.0118 & -0.0081 & -0.0090 & 0.3046 & 0.7204 \end{bmatrix}$$

$$\begin{bmatrix} 0.3043 & 0 & 0 & 0 & 0 \\ 0 & 0.2841 & 0 & 0 & 0 \\ 0 & 0 & 0.9323 & 0 & 0 \\ 0 & 0 & 0 & 0.9351 & 0 \\ 0 & 0 & 0 & 0 & 0.9344 \end{bmatrix}$$

$$\begin{bmatrix} -0.5750 & -0.1193 & -0.2770 & 0.6437 & 0.4050 \\ -0.8036 & -0.1008 & 0.1733 & -0.4822 & -0.2856 \\ 0.0220 & 0.0299 & -0.7986 & -0.5140 & 0.3109 \\ 0.1233 & -0.8086 & 0.2831 & -0.2053 & 0.4568 \\ -0.0892 & 0.5665 & 0.4188 & -0.2161 & 0.6701 \end{bmatrix}$$

Ca-Co-3(4)-LS-up(down)

$$\begin{bmatrix} 0.7349 & -0.3016 & 0.0036 & -0.0057 & 0.0062 \\ -0.3016 & 0.4811 & 0.0066 & 0.0059 & -0.0020 \\ 0.0036 & 0.0066 & 0.9315 & -0.0167 & 0.0117 \\ -0.0057 & 0.0059 & -0.0167 & 0.5303 & 0.3017 \\ 0.0062 & -0.0020 & 0.0117 & 0.3017 & 0.7092 \end{bmatrix}$$

$$\begin{bmatrix} 0.2807 & 0 & 0 & 0 & 0 \\ 0 & 0.3042 & 0 & 0 & 0 \\ 0 & 0 & 0.9355 & 0 & 0 \\ 0 & 0 & 0 & 0.9345 & 0 \\ 0 & 0 & 0 & 0 & 0.9321 \end{bmatrix}$$

$$\begin{bmatrix} -0.5527 & -0.0276 & 0.7803 & -0.2808 & 0.0776 \\ -0.8329 & -0.0124 & -0.5190 & 0.1882 & -0.0371 \\ 0.0123 & -0.0322 & -0.1577 & -0.1920 & 0.9680 \\ 0.0225 & -0.8005 & 0.1781 & 0.5604 & 0.1132 \\ -0.0120 & 0.5977 & 0.2553 & 0.7313 & 0.2067 \end{bmatrix}$$

Sr-Co-1-LS-up(down)

$$\begin{bmatrix} 0.7052 & -0.3045 & -0.0020 & 0.0015 & -0.0043 \\ -0.3045 & 0.5282 & -0.0045 & -0.0035 & -0.0012 \\ -0.0020 & -0.0045 & 0.9323 & 0.0189 & -0.0115 \\ 0.0015 & -0.0035 & 0.0189 & 0.4922 & 0.3040 \\ -0.0043 & -0.0012 & -0.0115 & 0.3040 & 0.7255 \end{bmatrix}$$

$$\begin{bmatrix} 0.2996 & 0 & 0 & 0 & 0 \\ 0 & 0.2824 & 0 & 0 & 0 \\ 0 & 0 & 0.9316 & 0 & 0 \\ 0 & 0 & 0 & 0.9345 & 0 \\ 0 & 0 & 0 & 0 & 0.9354 \end{bmatrix}$$

$$\begin{bmatrix} 0.5998 & -0.0249 & 0.3997 & -0.6596 & 0.2116 \\ 0.7994 & -0.0223 & -0.2960 & 0.4938 & -0.1704 \\ 0.0064 & -0.0342 & -0.7687 & -0.2736 & 0.5770 \\ 0.0308 & 0.8231 & 0.2085 & 0.2688 & 0.4537 \\ -0.0135 & -0.5659 & 0.3439 & 0.4171 & 0.6224 \end{bmatrix}$$

Sr-Co-2-LS-up(down)

$$\begin{bmatrix} 0.70577 & -0.3040 & 0.0065 & -0.0086 & 0.0089 \\ -0.3040 & 0.5285 & 0.0100 & 0.0085 & -0.0027 \\ 0.0065 & 0.0100 & 0.9319 & 0.0177 & -0.0107 \\ -0.0086 & 0.0085 & 0.0177 & 0.4930 & 0.3044 \\ 0.0089 & -0.0027 & -0.0107 & 0.3044 & 0.7256 \end{bmatrix}$$

$$\begin{bmatrix} 0.3003 & 0 & 0 & 0 & 0 \\ 0 & 0.2821 & 0 & 0 & 0 \\ 0 & 0 & 0.9314 & 0 & 0 \\ 0 & 0 & 0 & 0.9346 & 0 \\ 0 & 0 & 0 & 0 & 0.9353 \end{bmatrix}$$

$$\begin{bmatrix} 0.5983 & -0.0464 & -0.3689 & 0.6569 & -0.2688 \\ 0.7996 & -0.0239 & 0.2607 & -0.4936 & 0.2201 \\ -0.0173 & 0.0326 & -0.7971 & -0.1963 & 0.5699 \\ -0.0423 & -0.8216 & 0.2150 & 0.2829 & 0.4438 \\ 0.0224 & 0.5668 & 0.3383 & 0.4542 & 0.5979 \end{bmatrix}$$

Sr-Co-3(4)-LS-up(down)

$$\begin{bmatrix} 0.7264 & -0.3044 & 0.0020 & -0.0040 & 0.0025 \\ -0.3044 & 0.4895 & 0.0033 & 0.0022 & -0.0022 \\ 0.0020 & 0.0033 & 0.9311 & -0.0173 & 0.0125 \\ -0.0040 & 0.0022 & -0.0173 & 0.5253 & 0.3039 \\ 0.0025 & -0.0022 & 0.0125 & 0.3039 & 0.7097 \end{bmatrix}$$

$$\begin{bmatrix} 0.2813 & 0 & 0 & 0 & 0 \\ 0 & 0.2991 & 0 & 0 & 0 \\ 0 & 0 & 0.9346 & 0 & 0 \\ 0 & 0 & 0 & 0.9351 & 0 \\ 0 & 0 & 0 & 0 & 0.9318 \end{bmatrix}$$

$$\begin{bmatrix} -0.5646 & -0.0088 & -0.8181 & -0.1090 & -0.0089 \\ -0.8254 & 0.0030 & 0.5599 & 0.0729 & 0.0009 \\ 0.0060 & -0.0337 & 0.0175 & -0.0785 & -0.9962 \\ 0.0012 & -0.8027 & -0.0709 & 0.5918 & -0.0207 \\ -0.0021 & 0.5953 & -0.1094 & 0.7915 & -0.0844 \end{bmatrix}$$

In the hexagonally arranged IS-LS mixed-state, there are 5 inequivalent Co sites in. $Bi_2Ba_2Co_2O_8$ They are Co-1-IS, Co-1-LS, Co-2-IS, Co-2-LS, and Co-3-LS (Co-4-LS). Each Co site has two sets of matrices for spin-up and spin-down components. The detailed matrices are listed below:

Ba-Co-1-IS-up

$$\begin{bmatrix} 0.7193 & -0.2213 & 0.0080 & 0.0057 & 0.0051 \\ -0.2213 & 0.7086 & 0.0118 & 0.0056 & 0.0050 \\ 0.0080 & 0.0118 & 0.9436 & 0.0026 & 0.0025 \\ 0.0057 & 0.0056 & 0.0026 & 0.9484 & -0.0054 \\ 0.0051 & 0.0050 & 0.0025 & -0.0054 & 0.9452 \end{bmatrix}$$

$$\begin{bmatrix} 0.4919 & 0 & 0 & 0 & 0 \\ 0 & 0.9343 & 0 & 0 & 0 \\ 0 & 0 & 0.9393 & 0 & 0 \\ 0 & 0 & 0 & 0.9471 & 0 \\ 0 & 0 & 0 & 0 & 0.9526 \end{bmatrix}$$

$$\begin{bmatrix} -0.6979 & -0.6612 & 0.2590 & 0.0930 & 0.0028 \\ -0.7152 & 0.6379 & -0.2468 & -0.1429 & -0.0145 \\ 0.0309 & -0.3316 & -0.4711 & -0.8112 & -0.0954 \\ 0.0176 & 0.1379 & 0.4974 & -0.2482 & -0.8196 \\ 0.0157 & 0.1638 & 0.6346 & -0.5013 & 0.5648 \end{bmatrix}$$

Ba-Co-1-IS-down

$$\begin{bmatrix} 0.4057 & -0.2544 & 0.0325 & 0.0109 & 0.0122 \\ -0.25444 & 0.8007 & -0.0800 & 0.0054 & -0.0062 \\ 0.0325 & -0.0800 & 0.0874 & -0.0725 & -0.0383 \\ 0.0109 & 0.0054 & -0.0725 & 0.4639 & 0.3819 \\ 0.0122 & -0.0062 & -0.0383 & 0.3819 & 0.6196 \end{bmatrix}$$

$$\begin{bmatrix} 0.0613 & 0 & 0 & 0 & 0 \\ 0 & 0.1622 & 0 & 0 & 0 \\ 0 & 0 & 0.2811 & 0 & 0 \\ 0 & 0 & 0 & 0.9341 & 0 \\ 0 & 0 & 0 & 0 & 0.9387 \end{bmatrix}$$

$$\begin{bmatrix} -0.0276 & 0.0212 & -0.8987 & -0.4278 & -0.0899 \\ 0.0886 & 0.0603 & -0.4354 & 0.8837 & 0.1343 \\ 0.9382 & 0.3179 & 0.0258 & -0.1139 & 0.0713 \\ 0.3022 & -0.7128 & -0.0143 & 0.1061 & -0.6239 \\ -0.1408 & 0.6220 & 0.0434 & 0.1087 & -0.7613 \end{bmatrix}$$

Ba-Co-1-LS-up

$$\begin{bmatrix} 0.4967 & -0.2723 & -0.0259 & -0.0828 & -0.0938 \\ -0.2723 & 0.7631 & -0.0479 & -0.0155 & -0.0787 \\ -0.0259 & -0.0479 & 0.6534 & 0.3092 & -0.1493 \\ -0.0828 & -0.0155 & 0.3092 & 0.5843 & 0.1367 \\ -0.0938 & -0.0787 & -0.1493 & 0.1367 & 0.8479 \end{bmatrix}$$

$$\begin{bmatrix} 0.2925 & 0 & 0 & 0 & 0 \\ 0 & 0.2349 & 0 & 0 & 0 \\ 0 & 0 & 0.9338 & 0 & 0 \\ 0 & 0 & 0 & 0.9438 & 0 \\ 0 & 0 & 0 & 0 & 0.9404 \end{bmatrix}$$

$$\begin{bmatrix} 0.8086 & -0.1607 & 0.5410 & 0.1639 & 0.0297 \\ 0.4868 & -0.1728 & -0.6756 & -0.2575 & -0.4587 \\ -0.0893 & -0.6338 & 0.1335 & -0.6811 & 0.3296 \\ 0.2995 & 0.6503 & -0.1585 & -0.4146 & 0.5389 \\ 0.1079 & -0.3461 & -0.4560 & 0.5206 & 0.6242 \end{bmatrix}$$

Ba-Co-1-LS-down

$$\begin{bmatrix} 0.6900 & -0.2970 & 0.0582 & 0.0631 & 0.0774 \\ -0.2970 & 0.57644 & 0..0686 & 0.0539 & 0.1018 \\ 0.0582 & 0.0686 & 0.9166 & -0.0835 & 0.0304 \\ 0.0631 & 0.0539 & -0.0835 & 0.4441 & 0.2968 \\ 0.0774 & 0.1018 & 0.0304 & 0.2968 & 0.7116 \end{bmatrix}$$

$$\begin{bmatrix} 0.2829 & 0 & 0 & 0 & 0 \\ 0 & 0.2380 & 0 & 0 & 0 \\ 0 & 0 & 0.9335 & 0 & 0 \\ 0 & 0 & 0 & 0.9439 & 0 \\ 0 & 0 & 0 & 0 & 0.9405 \end{bmatrix}$$

$$\begin{bmatrix} -0.5883 & 0.1722 & 0.5906 & 0.3386 & -0.4010 \\ -0.7192 & 0.1758 & -0.5757 & 0.0683 & 0.3402 \\ 0.1033 & -0.1564 & 0.3249 & 0.5638 & 0.7358 \\ -0.1018 & -0.8347 & -0.2704 & 0.3500 & -0.3119 \\ 0.3401 & 0.4672 & -0.3755 & 0.6636 & -0.2910 \end{bmatrix}$$

Ba-Co-2-IS-up

$$\begin{bmatrix} 0.7200 & -0.2217 & -0.0069 & -0.0077 & -0.0035 \\ -0.2217 & 0.7072 & -0.0103 & -0.0079 & -0.0033 \\ -0.0069 & -0.0103 & 0.9440 & 0.0022 & 0.0028 \\ -0.0077 & -0.0079 & 0.0022 & 0.9492 & -0.0059 \\ -0.0038 & -0.0033 & 0.0028 & -0.0059 & 0.9454 \end{bmatrix}$$

$$\begin{bmatrix} 0.4912 & 0 & 0 & 0 & 0 \\ 0 & 0.9347 & 0 & 0 & 0 \\ 0 & 0 & 0.9392 & 0 & 0 \\ 0 & 0 & 0 & 0.9472 & 0 \\ 0 & 0 & 0 & 0 & 0.9536 \end{bmatrix}$$

$$\begin{bmatrix} -0.6962 & -0.6755 & -0.2289 & -0.0818 & 0.0030 \\ -0.7169 & 0.6511 & 0.2141 & 0.1265 & 0.0182 \\ -0.0268 & 0.2907 & -0.4825 & -0.8243 & -0.0497 \\ -0.0242 & -0.1102 & 0.4852 & -0.2724 & -0.8232 \\ -0.0107 & -0.1523 & 0.6585 & -0.4728 & 0.5653 \end{bmatrix}$$

Ba-Co-2-IS-down

$$\begin{bmatrix} 0.4047 & -0.2549 & -0.0282 & -0.0102 & -0.0105 \\ -0.2549 & 0.8033 & 0.0704 & -0.0019 & 0.0028 \\ -0.0282 & 0.0704 & 0.0849 & -0.0715 & -0.0380 \\ -0.0102 & -0.0019 & -0.0715 & 0.4642 & 0.3824 \\ -0.0105 & 0.0028 & -0.0380 & 0.3824 & 0.6190 \end{bmatrix}$$

$$\begin{bmatrix} 0.0610 & 0 & 0 & 0 & 0 \\ 0 & 0.1619 & 0 & 0 & 0 \\ 0 & 0 & 0.2803 & 0 & 0 \\ 0 & 0 & 0 & 0.9343 & 0 \\ 0 & 0 & 0 & 0 & 0.9386 \end{bmatrix}$$

$$\begin{bmatrix} 0.0220 & -0.0134 & 0.8995 & -0.4288 & -0.0801 \\ -0.0803 & -0.0451 & 0.4351 & 0.8877 & 0.1193 \\ 0.9394 & 0.3188 & 0.0228 & 0.0999 & -0.0739 \\ 0.3010 & -0.7129 & -0.0053 & -0.0906 & 0.6268 \\ -0.1416 & 0.6228 & 0.0330 & -0.0998 & 0.7622 \end{bmatrix}$$

Ba-Co-2-LS-up

$$\begin{bmatrix} 0.4934 & -0.2720 & 0.0275 & 0.0790 & 0.0955 \\ -0.2720 & 0.7665 & 0.0424 & 0.0200 & 0.0754 \\ 0.0275 & 0.0424 & 0.6516 & 0.3111 & -0.1477 \\ 0.0790 & 0.0200 & 0.3111 & 0.5830 & 0.1347 \\ 0.0955 & 0.0754 & -0.1477 & 0.1347 & 0.8500 \end{bmatrix}$$

$$\begin{bmatrix} 0.2914 & 0 & 0 & 0 & 0 \\ 0 & 0.2350 & 0 & 0 & 0 \\ 0 & 0 & 0.9341 & 0 & 0 \\ 0 & 0 & 0 & 0.9439 & 0 \\ 0 & 0 & 0 & 0 & 0.9401 \end{bmatrix}$$

$$\begin{bmatrix} 0.8129 & -0.1526 & -0.5404 & -0.1447 & -0.0546 \\ 0.4885 & 0.1522 & 0.6623 & 0.2472 & 0.4883 \\ 0.0790 & 0.6371 & 0.0856 & -0.6784 & 0.3469 \\ -0.2847 & -0.6576 & -0.1902 & -0.3988 & 0.5397 \\ -0.1153 & 0.3394 & -0.4752 & 0.5466 & 0.5891 \end{bmatrix}$$

Ba-Co-2-LS-down

$$\begin{bmatrix} 0.6936 & -0.2973 & -0.0557 & -0.0629 & -0.0763 \\ -0.2973 & 0.5710 & -0.0670 & -0.0538 & -0.1025 \\ -0.0557 & -0.0670 & 0.9178 & -0.0827 & 0.0312 \\ -0.0629 & -0.0538 & -0.0827 & 0.4471 & 0.2981 \\ -0.0763 & -0.1025 & 0.0312 & 0.2981 & 0.7089 \end{bmatrix}$$

$$\begin{bmatrix} 0.2815 & 0 & 0 & 0 & 0 \\ 0 & 0.2388 & 0 & 0 & 0 \\ 0 & 0 & 0.9339 & 0 & 0 \\ 0 & 0 & 0 & 0.9439 & 0 \\ 0 & 0 & 0 & 0 & 0.9405 \end{bmatrix}$$

$$\begin{bmatrix} -0.5876 & 0.1580 & -0.5919 & -0.3637 & 0.3835 \\ -0.7281 & 0.1594 & 0.5805 & -0.0418 & -0.3251 \\ -0.1016 & 0.1524 & 0.2957 & 0.5499 & 0.7594 \\ 0.0804 & 0.8348 & -0.2749 & 0.3558 & -0.3074 \\ -0.3282 & -0.4791 & -0.3869 & 0.6610 & -0.2758 \end{bmatrix}$$

Ba-Co-3(4)-LS-up

$$\begin{bmatrix} 0.7545 & -0.2702 & 0.0703 & 0.0989 & 0.0506 \\ -0.2702 & 0.5091 & 0.0832 & 0.0664 & 0.1396 \\ 0.0703 & 0.0832 & 0.9010 & -0.1086 & 0.0467 \\ 0.0989 & 0.0664 & -0.1086 & 0.5216 & 0.2905 \\ 0.0506 & 0.1396 & 0.0467 & 0.2905 & 0.6537 \end{bmatrix}$$

$$\begin{bmatrix} 0.2476 & 0 & 0 & 0 & 0 \\ 0 & 0.2782 & 0 & 0 & 0 \\ 0 & 0 & 0.9314 & 0 & 0 \\ 0 & 0 & 0 & 0.9439 & 0 \\ 0 & 0 & 0 & 0 & 0.9389 \end{bmatrix}$$

$$\begin{bmatrix} 0.4246 & 0.2954 & -0.6774 & -0.5179 & 0.0736 \\ 0.5469 & 0.5774 & 0.6010 & 0.0031 & 0.0796 \\ -0.2384 & 0.0147 & 0.2921 & -0.6613 & -0.6483 \\ -0.6366 & 0.4562 & 0.0690 & -0.2733 & 0.5543 \\ 0.2419 & -0.6091 & 0.2998 & -0.4688 & 0.5106 \end{bmatrix}$$

Ba-Co-3(4)-LS-down

$$\begin{bmatrix} 0.5482 & -0.2900 & -0.0971 & -0.0760 & 0.1016 \\ -0.2900 & 0.7178 & -0.0638 & -0.0737 & -0.0742 \\ -0.0971 & -0.0638 & 0.7687 & 0.2405 & -0.1344 \\ -0.0760 & -0.0737 & 0.2405 & 0.4706 & 0.1687 \\ -0.1016 & -0.0742 & -0.1344 & 0.1687 & 0.8361 \end{bmatrix}$$

$$\begin{bmatrix} 0.2483 & 0 & 0 & 0 & 0 \\ 0 & 0.2746 & 0 & 0 & 0 \\ 0 & 0 & 0.9338 & 0 & 0 \\ 0 & 0 & 0 & 0.9439 & 0 \\ 0 & 0 & 0 & 0 & 0.9408 \end{bmatrix}$$

$$\begin{bmatrix} 0.4673 & 0.5984 & -0.6298 & -0.1127 & -0.1189 \\ 0.3297 & 0.4667 & 0.6690 & 0.3916 & -0.2695 \\ 0.4783 & -0.1449 & 0.0089 & 0.3110 & 0.8084 \\ -0.5423 & 0.6325 & 0.1364 & -0.1838 & 0.5035 \\ 0.3874 & -0.0548 & 0.3703 & -0.8387 & 0.0796 \end{bmatrix}$$
